# Calcium Mechanisms in Limb-Girdle Muscular Dystrophy with *CAPN3* Mutations

**DOI:** 10.3390/ijms20184548

**Published:** 2019-09-13

**Authors:** Jaione Lasa-Elgarresta, Laura Mosqueira-Martín, Neia Naldaiz-Gastesi, Amets Sáenz, Adolfo López de Munain, Ainara Vallejo-Illarramendi

**Affiliations:** 1 Biodonostia, Neurosciences Area, Group of Neuromuscular Diseases, 20014 San Sebastian, Spain; Jaione.lasa@biodonostia.org (J.L.-E.); Laura.mosqueira@biodonostia.org (L.M.-M.); Neia.naldaiz@biodonostia.org (N.N.-G.); MIRENAMETSA.SAENZPENA@osakidetza.eus (A.S.); 2 CIBERNED, Instituto de Salud Carlos III, Ministry of Science, Innovation and Universities, 28031 Madrid, Spain; 3 Departmento de Neurosciencias, Universidad del País Vasco UPV/EHU, 20014 San Sebastian, Spain; 4 Osakidetza Basque Health Service, Donostialdea Integrated Health Organisation, Neurology Department, 20014 San Sebastian, Spain; 5 Grupo Neurociencias, Departmento de Pediatría, Hospital Universitario Donostia, UPV/EHU, 20014 San Sebastian, Spain

**Keywords:** calpain 3, calcium, LGMD2A, LGMDR1, muscular dystrophies, calpainopathy

## Abstract

Limb-girdle muscular dystrophy recessive 1 (LGMDR1), previously known as LGMD2A, is a rare disease caused by mutations in the *CAPN3* gene. It is characterized by progressive weakness of shoulder, pelvic, and proximal limb muscles that usually appears in children and young adults and results in loss of ambulation within 20 years after disease onset in most patients. The pathophysiological mechanisms involved in LGMDR1 remain mostly unknown, and to date, there is no effective treatment for this disease. Here, we review clinical and experimental evidence suggesting that dysregulation of Ca^2+^ homeostasis in the skeletal muscle is a significant underlying event in this muscular dystrophy. We also review and discuss specific clinical features of LGMDR1, CAPN3 functions, novel putative targets for therapeutic strategies, and current approaches aiming to treat LGMDR1. These novel approaches may be clinically relevant not only for LGMDR1 but also for other muscular dystrophies with secondary calpainopathy or with abnormal Ca^2+^ homeostasis, such as LGMD2B/LGMDR2 or sporadic inclusion body myositis.

## 1. Overview of Calcium Homeostasis in the Skeletal Muscle

**Figure 1:**
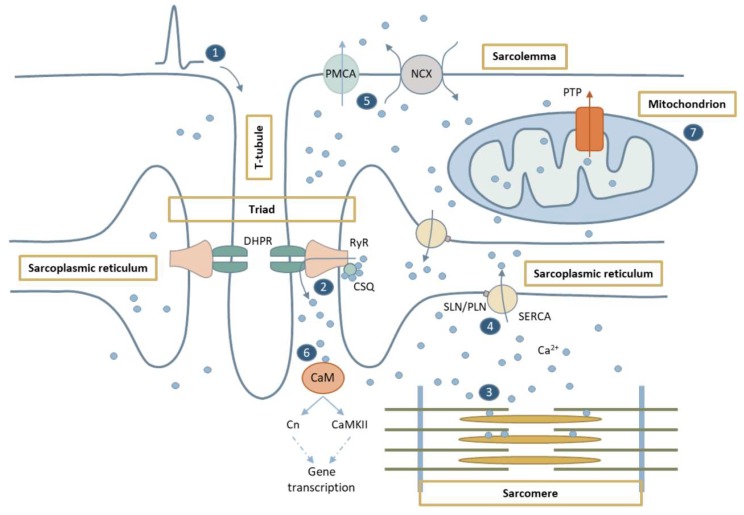
Representation of Ca^2+^ fluxes in the muscle fiber. Upon sarcolemmal depolarization reaching T-tubules (1), DHPRs undergo a conformational change that activates RyR1 channels and results in Ca^2+^ release from the SR (2). Ca^2+^ diffuses to the sarcomere where it initiates muscle contraction (3). Muscle relaxation takes place when Ca^2+^ is sequestered into the SR by SERCAs (4) or pumped out of the fiber by membrane channels (NCX, PMCA) (5). Cytosolic Ca^2+^ also binds CaM, which activates the Ca^2+^-dependent signaling pathways resulting in muscle gene regulation (6). Cytosolic Ca^2+^ also reaches mitochondria (7), where it stimulates metabolism and ATP synthesis required for muscle contraction and relaxation.

Ca^2+^ plays a vital role in a wide range of cellular processes such as gene transcription, membrane resealing, secretion, neurotransmission, as well as cell differentiation, proliferation, or survival [1,2]. In skeletal muscle fibers, Ca^2+^ is crucial for both electric activation along the motor endplate and skeletal muscle contraction. In addition, Ca^2+^ is involved in many other functions such as protein synthesis, protein degradation, fiber type shifting, Ca^2+^-regulated proteolysis, transcription factor modulation, mitochondrial adaptation, cell plasticity, and respiration [3]. Therefore, tight regulation of Ca^2+^ levels is essential for the proper function of skeletal muscle (Figure 1).

### 1.1. Ca^2+^ in Excitation-Contraction Coupling

Muscle contraction initiates by depolarization of the sarcolemma in response to acetylcholine release from motoneurons. The action potential propagates into the triads, which are anatomical structures formed by the association of sarcolemmal transverse tubules (T-tubules) and sarcoplasmic reticulum (SR) terminal cisternae [4]. Triads play an essential role in excitation-contraction coupling (ECC) since they allow close contact and synchronization between crucial receptors in the sarcolemma and the SR. See [3,5] for review. Membrane depolarization activates dihydropyridine receptors (DHPRs) in the T-tubules [6], and this results in activation of the closely apposed ryanodine receptors (RyRs), the main Ca^2+^ release channels in the SR [7]. RyR1, the predominant RyR isoform in skeletal muscle, releases Ca^2+^ from the SR into the cytosol either after activation by DHPRs or increased cytosolic Ca^2+^ levels [8]. RyR1 function is modulated by post-translational modifications, such as S-nitrosylation, S-glutathionylation, and phosphorylation by both Protein Kinase A (PKA) and Ca^2+^/calmodulin-dependent protein kinase II (CaMKII) [9]. RyRs operate in coordination with other proteins in order to maintain the balance between Ca^2+^ release, Ca^2+^ storage, and Ca^2+^ reuptake [10]. Indeed, a variety of proteins and small molecules, both in the SR lumen and cytosol are needed for this tight coordination [9]. On the cytosolic side, calmodulin (CaM), a Ca^2+^ sensor, has a dual effect on RyR1, functioning as an activator at low cytosolic Ca^2+^ levels and as an inhibitor at high cytosolic Ca^2+^ levels [11]. On the SR luminal side, calsequestrin (CSQ) forms a complex with RyRs, junctin, and triadin. RyR function is inhibited by the binding of CSQ, which essentially depends on luminal Ca^2+^ concentration [12]. CSQ is the main Ca^2+^-binding protein in the SR lumen, and it functions as an endogenous regulator of Ca^2+^ fluxes and as a Ca^2+^ reservoir with a moderate affinity but high capacity to bind Ca^2+^ [13]. After Ca^2+^ release into the cytosol through RyR1, Ca^2+^ binds to various cytosolic Ca^2+^ buffers, such as ATP and CaM, and it can also be sequestered by mitochondria. At the sarcomere, the contractile unit of the skeletal muscle, troponin C undergoes a Ca^2+^-dependent conformational change that ultimately results in myosin and actin cross-bridge cycling and muscle contraction [14]. Upon muscle excitation, cytosolic Ca^2+^ levels raise from ~100 nM (resting levels) to ~10µM in slow fibers, and ~18 µM in fast fibers [15].

Muscle relaxation initiates by lowering of cytosolic Ca^2+^ back to resting levels, which mainly relies on sarco/endoplasmic reticulum Ca^2+^ ATPase (SERCA) pumps [16], and sarcolemmal Ca^2+^ transporters such as Na^+^/Ca^2+^ exchangers (NCX1-3) and the plasma membrane Ca^2+^-ATPase (PMCA) [3]. SERCA actively transports Ca^2+^ from the cytosol into the SR against a large concentration gradient at the expense of ATP hydrolysis. Fast muscle fibers express SERCA1a isoform, whereas slow fibers and cardiac muscle express SERCA2a isoform [16]. Since SERCA pumps are major ATP consumers, they are strongly affected by changes in cell energetics and ATP supply [17]. Similarly to RyRs, SERCAs are also modulated by several cytosolic and SR luminal proteins, as well as by post-translational modifications, including N-glycosylation, S-glutathionylation, and phosphorylation [18], although there is some controversy over SERCA modulation through phosphorylation. Also, the short integral membrane proteins phospholamban (PLN) and sarcolipin (SLN) inhibit SERCA activity [19]. Phosphorylation of PLN by PKA or CaMKII results in SERCA activation through dissociation of PLN from the Ca^2+^ pump [18].

### 1.2. Ca^2+^-Mediated Signaling Pathways

In addition to its role in ECC, Ca^2+^ is also a key regulator of gene transcription triggered by different stimuli. For instance, intracellular Ca^2+^ signals mediate transcriptional changes necessary for skeletal muscle adaptation in response to changes in activation patterns [20]. CaMK pathway and Calcineurin (Cn), a Ca^2+^/CaM-dependent serine/threonine protein phosphatase, have crucial roles in many of these Ca^2+^-mediated signaling processes. Cn stimulates the transcription of both NFAT and NF-κB targeted genes [21,22]. While the Cn/NFAT pathway responds preferentially to sustained and low-amplitude elevations of intracellular Ca^2+^, high amplitude oscillations activate NF-κB [23]. NFAT signaling induces the slow gene program during muscle regeneration and maintains the slow fiber phenotype in the adult muscle tissue, whereas NF-κB regulates the gene program for myoblast proliferation and differentiation [24,25]. Interestingly, depending on the stimulus nature, NF-κB can behave either as a promoter or antagonist of apoptosis [26].

CaMK pathway is involved in the regulation of contraction-induced Ca^2+^ handling, and mitochondrial biogenesis. It also regulates gene expression in skeletal muscle, promoting the slow to fast fiber shift [27]. Also, during muscle development and adaptation, CaMK activates critical transcription factors such as MEF2 (myocyte enhancer factor 2) via phosphorylation of class II histone deacetylases (HDACs) [28]. CaMKII, the main CaMK in the skeletal muscle, is involved in the maintenance of myofiber phenotype and muscle growth [29,30]. In addition, CaMKII is one of the upstream activators of AMP-activated protein kinase (AMPK), an energy sensor that coordinates cell growth and autophagy, as well as regulates mitochondrial function and biogenesis. Under low intracellular ATP levels, AMPK promotes catabolic pathways to generate more ATP and suppresses anabolic pathways such as the high ATP consuming mTORC1 pathway, to inhibit cell growth [31].

### 1.3. Ca^2+^ in Mitochondria

Mitochondria are, together with SR, main Ca^2+^ storages, and they also function as a source of intracellular Ca^2+^ [2]. They account for about 15% of the cytosolic volume in oxidative fibers [32], and they are located close to the SR in the skeletal muscle. There is a significant interplay between mitochondria and SR through the mitochondria-associated SR membrane (MAM), which is essential for cell physiology and Ca^2+^ homeostasis [33]. Ca^2+^ regulates mitochondrial metabolism, biogenesis, motility, distribution, and plasticity [34,35,36]. In turn, mitochondria can also affect intracellular Ca^2+^ levels. Thus, ECC triggers transient Ca^2+^ increases in the mitochondrial matrix, which are essential to promote mitochondrial metabolism and ATP synthesis. This is required to balance the ATP consumption of actomyosin cross-bridge cycling and SERCA pumps during contraction and relaxation, respectively [37]. Alternatively, Ca^2+^ overload in the mitochondria may induce the opening of permeability transient pores (PTP) that results in a massive release of Ca^2+^ and pro-apoptotic factors such as cytochrome C (Cyt-c) into the cytosol, which, in turn, activate caspase 9 and initiate the apoptotic program [38,39].

Ca^2+^ is also involved in mitochondrial biogenesis through regulation of the CaMKII pathway. Indeed, the expression of peroxisome proliferator-activated receptor gamma coactivator 1 alpha (PGC1α), a key regulator of mitochondrial biogenesis, depends on the Ca^2+^-mediated activation of CaMKII [40]. Finally, mitochondrial motility also relies on cytosolic [Ca^2+^]. Indeed, myosin-Va, a Ca^2+^ sensor molecule, may regulate mitochondria-bound molecular motors allowing mitochondrial movements along cytoskeletal fibers and controlling the distribution of mitochondria to enhance Ca^2+^ buffering and ATP production in regions with high cytosolic [Ca^2+^] [36].

## 2. Limb-Girdle Muscular Dystrophy-Recessive 1

LGMDR1, also known as calpainopathy and previously symbolized LGMD2A [41], is caused by homozygous or compound heterozygous mutation in the *CAPN3* gene encoding for the proteolytic enzyme calpain 3 (CAPN3) [42]. Recently, heterozygous mutations in the *CAPN3* gene have been reported to cause autosomal dominant limb-girdle muscular dystrophy-4 (LGMDD4), with a later onset and milder phenotype [43,44,45], although the mechanisms underlying these cases still need some clarification [46]. The prevalence of LGMDR1 ranges from 1 to 9 cases per 100,000 people, and it represents almost 30% of all LGMD cases in open populations [47,48,49,50,51,52,53] with some ancestral mutations responsible for specific ethnic or geographic clusters [54,55,56,57,58]. By September 2019, there are more than 480 pathogenic variants of *CAPN3* reported in the Leiden Open Variation database [59]. The molecular spectrum covers all *CAPN3* exons with some hot regions related to severe or benign phenotypes, as well as intronic variants [51,60].

LGMDR1 is characterized by progressive muscle weakness and degeneration, with a predominant effect on shoulder, pelvic, and proximal limb muscles [61]. There is no affection of cardiac and facial muscles, and no cognitive defects have been reported in this disease [47]. Age of onset is highly variable, although initial symptoms usually appear between eight and 15 years [62] and patients loss ambulation around 10 to 20 years after the onset [54,55]. However, lately, benign forms are being increasingly reported with preserved ambulation even after reaching 60 or more years old. In general, these benign forms have metabolic symptoms at onset (myalgia, cramps, and exercise intolerance) or even asymptomatic hyper-creatine kinase-emia that may carry on for years before muscle weakness. Symptoms of the classical LGMDR1 phenotype fit with the criteria described by Erb in 1884 to define juvenile muscular dystrophy [54]. However, there is certain variability regarding disease progression and severity related to gender, as well as the type and localization of mutations [51]. Moreover, a phenomenon known as de novo intermolecular complementation (iMOC) of CAPN3 may also lead to a milder phenotype in compound heterozygotes [63]. Additionally, in some families, there is considerable phenotypic variability among patients with identical mutations [64], which makes prognosis in LGMDR1 very challenging [47].

**Figure 2:**
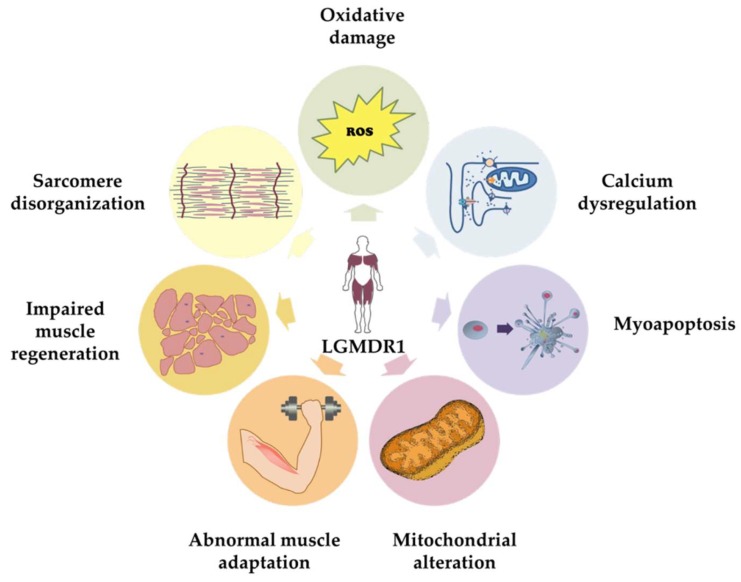
Illustration of the pathological features of CAPN3 deficiency in the skeletal muscle.

**Figure 3:**
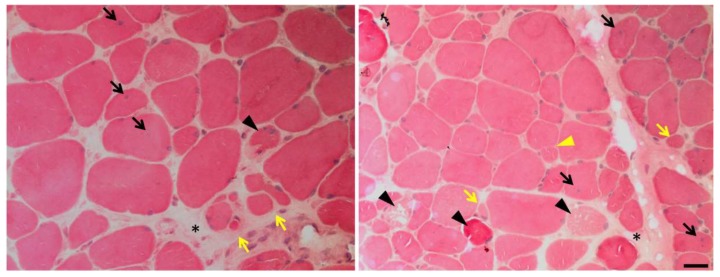
Muscle biopsy of a LGMDR1 patient. Hematoxylin and eosin staining shows endomysial fibrosis (black asterisks), central nuclei (black arrows), fiber splitting (yellow triangle), necrosis (black triangles), atrophic fibers (yellow arrows) and increased variation in fiber size and shape. Scale bar: 25 μm.

Since the discovery of *CAPN3* as the gene responsible for LGMDR1, several groups have been trying to identify the pathogenic mechanisms that may give rise to the clinical and histological features of LGMDR1. Although to date, these mechanisms are not entirely understood, there is solid evidence indicating that CAPN3 is a multifunctional protein. Different studies performed in animal models and human samples have shown that CAPN3 deficiency is associated with different features in the skeletal muscle such as oxidative damage [65,66], Ca^2+^ dysregulation [67,68], sarcomere disorganization [69], mitochondrial abnormalities [66,70,71,72], abnormal muscle adaptation [73,74], and impaired muscle regeneration [71], which together would lead to inflammation, necrosis, fibrosis, atrophy, and progressive muscle degeneration, characteristic of LGMDR1 (Figure 2 and Figure 3). Indeed, patients in the early stages of the disease present an increased concentration of serum creatine kinase (CK), which is an unspecific hallmark of muscle damage [55,75]. Some patients at this stage present eosinophilic infiltrations associated with peripheral blood eosinophilia that have an unclear pathogenic significance [50,54,76]. Fibrosis is often present, and it tends to increase with disease progression [75].

Muscle biopsies from LGMDR1 patients also present general dystrophic features, such as necrotic areas with regenerative regions, fiber size variability, central nuclei, and disorganized myofibrils (Figure 3). Finally, muscles from LGMDR1 patients may present myonuclear apoptosis and a peculiar pattern of focal degeneration, as well as fibers with a lobulated pattern, commonly attributed to sarcomere and mitochondria disorganization [50].

## 3. CAPN3 Localization and Function

**Figure 4:**
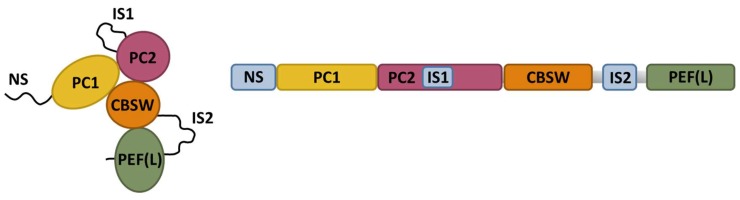
Schematic representation of CAPN3 structure. CAPN3 is comprised of two protease core domains (PC1 and PC2), a calpain-type β-sandwich domain (CBSW), and a penta E-F hand domain (PEF) that binds four calcium ions and may contribute to CAPN3 dimerization. The three specific regions of CAPN3 (NS, IS1, and IS2) are shown in blue. Schematics have been modified from Ye et al., 2018 [82]. The right panel depicts the estimated tertiary structure of CAPN3.

CAPN3 belongs to the calpain superfamily of Ca^2+^-dependent non-lysosomal cysteine proteases. Calpains have relevant functions in many cellular processes, including cell motility, apoptosis, cell differentiation, and cell-cycle regulation. In these processes, intracellular Ca^2+^ activates calpains, which then cleave specific substrates [77,78,79,80]. In mammals, the ubiquitous CAPN1 (µ-calpain) and CAPN2 (m-calpain) are the most extensively studied calpains. CAPN3 shares catalytic domain structure with CAPN1 and CAPN2 but CAPN3 has three unique regions, namely NS, IS1, and IS2, (Figure 4) that confer this protein some unusual features, such as an extreme instability and fast autodegradation rate, and a Na^+^-dependent activation [81].

One of the most remarkable features of CAPN3 is its extreme autodegradation rate [83], which has hindered conventional biochemical analysis. Indeed, the native structure of CAPN3 remains unresolved, as well as the accurate interactions of CAPN3 with other proteins. Another extremely unusual characteristic of CAPN3 is its ability to regain proteolytic function after its autolytic dissociation. This occurs through iMOC, a process where two autolytic fragments of CAPN3 reconstitute an active core protease domain [84]. With regards to its proteolytic function, CAPN3 can be activated at physiological intracellular concentrations of Ca^2+^ (100 nM) and Na^+^ (15 mM) [85], and thus, Na^+^ is responsible for the low Ca^2+^ levels required to activate CAPN3. In fact, in absence of Na^+^, the Ca^2+^ concentration required for CAPN3 autolysis would be around 0.1 mM. Hence, in the skeletal muscle, CAPN3 autolytic activity is suppressed in vivo, through its binding to connectin/titin [86]. CAPN3 presents both proteolytic as well as non-proteolytic functions [81]. Different studies have identified several mechanisms dependent on CAPN3 proteolytic function, such as mechanosensory transduction and sarcomere remodeling after exercise [81,87]. Non-proteolytic features of CAPN3, independent of its protease activity, have been identified through comparative studies in CAPN3 knockout and knock-in mice. These studies indicate that CAPN3 contributes to the maintenance of Ca^2+^ homeostasis through the stabilization of critical Ca^2+^-handling proteins, which relies on non-proteolytic functions of CAPN3 [88,89,90]. These functions will be further discussed in the following section.

CAPN3 presents a broad distribution within the muscle fiber, having been found at the sarcomere, membrane fraction, sarcoplasmic reticulum (SR), cytosol, and even the nucleus [88,91,92]. CAPN3 has been mainly implicated in the regulation of muscle contraction and sarcomere stability [85,92,93,94]. Within the sarcomere, CAPN3 is localized in several regions, where it interacts with different proteins. CAPN3 interacts at the Z-line with α-actinin-3, tropomyosin, and LIM-domain binding protein 3. CAPN3 also interacts with titin, a large scaffold protein that plays a vital role in sarcomere assembly and passive tension of myofibrils, as well as in mechanosensory transduction pathways [92,95]. Interestingly, CAPN3 binds to titin at the N2A and M-line regions, with different affinity depending on the sarcomere length. Thus, the presence of CAPN3 at the N2A region is increased compared to the M-line region when the sarcomere stretches. This location shift along different titin regions is facilitated by CAPN3 proteolytic activity [93], and it is crucial for the dissociation of Muscle Ankyrin Repeat Protein-2 (MARP-2) from titin, and its translocation to the nucleus to transmit signals of mechanical perturbation [96], which suggests that CAPN3 may function as a sensor of sarcomere integrity.

Titin stabilizes CAPN3 by preventing its autodegradation [69], and therefore, CAPN3 activity may be regulated by titin binding. On the other hand, since titin is also a CAPN3 substrate, CAPN3 may be responsible for the fast turnover of titin [97], and likely other sarcomere proteins, an aspect that is necessary for appropriate maintenance of the sarcomere structure [98,99]. In this line, C3KO mice present abnormal A-bands at the sarcomere and delayed myofibrillogenesis [69], together with an accumulation of high molecular weight ubiquitin-protein conjugates [87]. Altogether these findings suggest that proper interaction between CAPN3 and titin is essential for sarcomere maintenance and remodeling [100].

## 4. Ca^2+^-Mediated Pathogenic Mechanisms Involved in CAPN3 Deficiency

**Figure 5:**
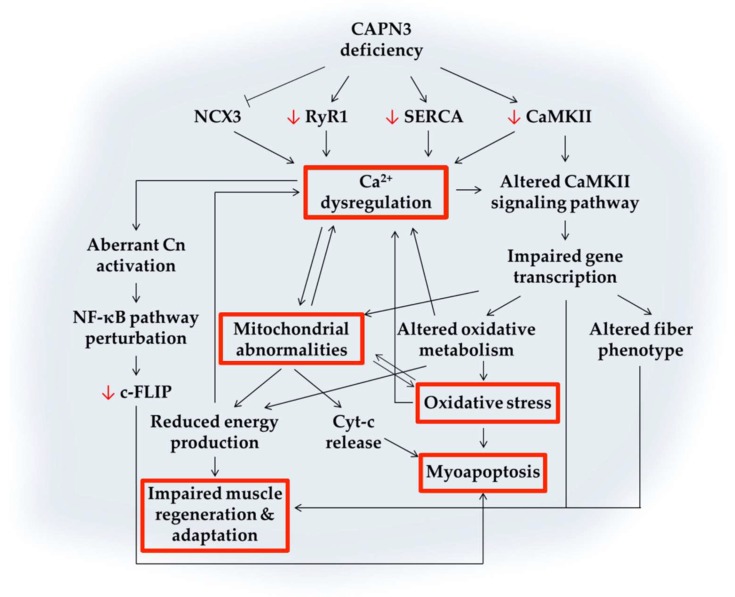
Schematic representation of putative Ca^2+^-mediated pathogenic mechanisms triggered by CAPN3 deficiency. CAPN3 deficiency results in reduced levels of RyR1, SERCA, and CaMKII. In addition, NCX3 activity may also be reduced. The decreased function of major Ca^2+^-handling proteins results in Ca^2+^ dysregulation and increased intracellular [Ca^2+^]. Reduced CaMKII levels together with Ca^2+^ dysregulation compromise CaMK downstream signaling pathways, which may lead to impaired gene transcription, mitochondrial abnormalities, oxidative stress, altered fiber phenotype, and impaired muscle regeneration. Mitochondrial abnormalities aggravate Ca^2+^ dysregulation and oxidative damage. They may also impact energy production and promote apoptosis through Cyt-c release and activation of caspases. Among these mechanisms, multiple feedback loops lead to altered Ca^2+^ levels and may result in myoapoptosis and muscle waste. Black arrows or blunt ends indicate enhancing or inhibitory effects, respectively. Red arrows indicate decreased protein expression. Text in red boxes represent several pathological features of CAPN3, as described in Figure 2.

Evidence obtained from animal models and human patients implicates Ca^2+^ homeostasis as a pathophysiological mechanism underlying different muscular dystrophies, including LGMDR1 [5]. Several mouse models of LGMDR1 have been used to understand the pathogenic mechanisms resulting from CAPN3 deficiency. Among these models, the CAPN3 knockout mouse lines Capn3^−/−^ and C3KO [69,101], and the CAPN3 knock-in mice Capn3^CS/CS^, expressing a structurally intact but inactive CAPN3 [96,102] have enabled to identify specifically non-proteolytic and proteolytic functions of CAPN3. Noteworthy, the phenotype of these mouse models does not fully recapitulate the severity of LGMDR1 in human patients, likely due to the higher regenerative potential of the murine muscle [103] and/or the increased Ca^2+^ buffering capacity of murine fibers [67]. Therefore, data from cellular models and muscle samples of patients with LGMDR1 need to be considered in order to unravel the molecular pathways involved in LGMDR1. During the last years, our group has been working on understanding these mechanisms using human biopsies as well as primary and immortalized human myotubes [67,68,104]. Below, we will review recent findings on pathogenic mechanisms associated with CAPN3 deficiency, with a particular focus on the involvement of Ca^2+^ dysregulation, as summarized in Figure 5.

### 4.1. Calcium Dysregulation

CAPN3 has been found to interact with several key Ca^2+^-handling proteins, such as RyR1, CSQ, and SERCA [88]. In this line, several studies have shown that CAPN3 deficiency results in abnormal Ca^2+^ handling in the skeletal muscle. Indeed, previous studies performed on the CAPN3 knockout mice C3KO and Capn3^−/−^, indicate that these mice present reduced RyR1 expression together with a decrease in SR Ca^2+^ release [89,90]. Also, Capn3^−/−^ knockout myotubes display reduced SR Ca^2+^ levels together with a lower response to SERCA inhibitors compared to wild-type myotubes [90]. Moreover, we have also contributed to a study showing reduced RyR1 expression and CaMKII signaling in muscles from LGMDR1 patients and C3KO mice [73]. At the triads, CAPN3 is part of a complex comprised of RyR1, AldoA, and CaMKII. Interestingly, in the absence of CAPN3, in C3KO mice, both RyR1 and CaMKII protein levels are decreased while AldoA is mislocalized. Thus, CAPN3 has been proposed as a structural stabilizer of RyR1 complexes at the triads [73,89]. This structural function of CAPN3 may depend on specific genetic regions since a recent study with LGMDR1 patients has reported a new CAPN3 mutation that does not result in diminished RyR1 in the skeletal muscle [70].

Our group has recently found that mouse and human myotubes deficient for CAPN3 display decreased SERCA protein levels as well as impaired Ca^2+^ reuptake into the SR [67,68]. Moreover, we also found reduced SERCA expression in muscle samples from LGMDR1 patients. In line with these findings, a recent study has shown that in sporadic inclusion body myositis, a common acquired muscle disease associated with aging, there is a secondary calpainopathy and a concomitant reduction of SERCA proteins that leads to Ca^2+^ dyshomeostasis [105]. Interestingly, we have found that SERCA deficiency in CAPN3 knockdown myotubes resulted in increased resting intracellular [Ca^2+^] in human myotubes, but not in mouse myotubes [67]. This is likely due to the fact that mouse muscle fibers have a higher Ca^2+^ buffer capacity, since parvalbumin, a major cytosolic Ca^2+^ buffer, is highly expressed in the mouse muscle [68]. In any case, SERCA mRNA levels were found unchanged in CAPN3 deficient samples, and therefore, we propose that CAPN3 is necessary to stabilize SERCA proteins and prevent their degradation, similarly to RyR1 and CaMKII. Interestingly, impaired SERCA function leads to SR Ca^2+^ depletion and ultimately to SR stress that activates the unfolded protein response (UPR) [106,107]. Indeed, in CAPN3 deficient myotubes, we found that several makers of SR stress and UPR are upregulated such as GRP78, CHOP, HERP, and the XBP1 spliced variant [68].

Finally, CAPN3 may also regulate Ca^2+^ homeostasis by increasing the activity of NCX3. In HEK293T Michel et al. describe an increased activity of the NCX3 muscle isoform, NCX3-AC, following CAPN3 cleavage [108]. This Na^+^/Ca^2+^ exchanger is found at the triads and extrudes Ca^2+^ across the sarcolemma to lower intracellular [Ca^2+^] during relaxation [108]. Nevertheless, more studies need to be performed in order to verify the regulation of NCX3 by CAPN3 in myotubes as well as in LGMDR1 models.

### 4.2. Abnormal Muscle Adaptation

The skeletal muscle is a remarkably adaptable tissue that responds to physiological and environmental challenges by altering its size and composition [109]. Ca^2+^ plays a fundamental role in muscle adaptation to changes in functional demand by activating specific Ca^2+^-dependent transcriptional pathways, such as CaMK pathways, in order to control muscle growth, fiber type transition, or mitochondrial biogenesis. In particular, CaMKII has been proposed as a major sensor of muscle activity that translates it into phenotypic adaptations by regulating the transcription of specific genes [30]. CaMKII signaling has been found severely compromised in the CAPN3 knockout mice, C3KO, and thus, abnormal muscle adaptation has been proposed as a major instrumental mechanism in LGMDR1 [73,74]. Indeed, in C3KO mice, reduced CaMKII expression results in an impaired slow myogenic program [73]. This may explain why muscles highly enriched in slow-twitch fibers, such as soleus and diaphragm are the most severely affected in C3KO mice, and also why there seems to be a preferential involvement of slow fibers in LGMDR1 muscle biopsies [73]. Moreover, after endurance exercise, CAPN3 deficient muscles fail to upregulate several groups of genes associated with muscle adaptation, such as myofibrillar, mitochondrial, and metabolic genes [74]. In this line, we have observed that human myotubes deficient for CAPN3 show reduced protein levels of CaMKII (unpublished data).

### 4.3. Mitochondrial Abnormalities

Several studies support the notion that CAPN3 is an important modulator of mitochondrial function, and its absence seems to have major consequences over this organelle [72,110]. LGMDR1 patients and CAPN3 knockdown mice present abundant mitochondria with abnormal spatial distribution [50,72,111], and a recent study points toward mutation-specific patterns of mitochondrial dysfunction in different LGMDR1 patients [70]. Moreover, in C3KO mice, mitochondria display a swollen appearance with disrupted membranes [72], while in LGMDR1 muscle biopsies, several mitochondrial genes have been found to be deregulated [112]. Mitochondrial abnormalities would have a direct impact on several pathomechanisms in LGMDR1, including Ca^2+^ dysregulation, energy deficits, oxidative stress, and ultimately, they could lead to cell death through the release of different pro-apoptotic factors into the cytosol (Figure 3). Indeed, C3KO muscles present decreased ATP production and increased oxidative stress [72], while LGMDR1 muscles show Cyt-c mislocalization to the cytosol [91], which may promote activation of caspases and apoptosis [113]. Likewise, mitochondria biogenesis and function are severely affected by Ca^2+^ dyshomeostasis [5]. Thus, while Ca^2+^ promotes mitochondrial metabolism and ATP synthesis, sustained high intracellular Ca^2+^ levels, such as the ones found in CAPN3 deficient myotubes [67,68], may result in mitochondrial Ca^2+^ overload and eventually lead to mitochondrial dysfunction and muscle cell degeneration. On the other hand, mitochondria biogenesis is modulated by PGC1α, which is in turn regulated by Cn and CaMK pathway, and therefore it is highly susceptible to Ca^2+^ dysregulation [40,114]. In this line, during muscle regeneration, CAPN3 knockout mice have proved unable to increase mitochondrial DNA content, as well as PGC1α and ATP5D transcripts, likely due to diminished CaMKII signaling [71].

### 4.4. Oxidative Stress

Oxidative and nitrosative stress have been associated with muscle wasting in several muscular dystrophies, including LGMDR1, where NAPDH oxidase appears to be one potential source of oxidative stress in LGMDR1 muscle biopsies [65]. In LGMDR1 biopsies, elevated reactive oxygen species (ROS), increased oxidized proteins, and lipid peroxidation have been reported [65,66]. Oxidative stress has also been associated with C3KO mouse muscles [72], where defective mitochondria may be a contributing factor since they are considered to generate the majority of cellular free radicals. Reciprocally, oxidative stress may also lead to mitochondrial damage [115], as well as Ca^2+^ dysregulation [116]. Therefore, identification of the cause-effect relationship among these features is very challenging. In any case, oxidative stress can cause cell death through necrotic or apoptotic pathways.

In response to oxidative stress, several protective mechanisms are activated to buffer extra ROS, such as superoxide dismutases (SOD). An increment in their concentration is indicative of oxidative stress. Accordingly, in CAPN3 knockout mouse increased levels of SOD have been reported, indicating a suitable protective response against oxidative stress [66]. In contrast, a reduction of antioxidant defense mechanisms has been described in LGMDR1 biopsies [66], pointing toward a higher vulnerability of human LGMDR1 muscles compared to mouse C3KO muscles.

### 4.5. Impaired Muscle Regeneration

Impaired muscle regeneration is another main pathological feature of LGMDR1 [71]. Muscle regeneration is a complex process that is initiated upon injury of muscle cells and can be divided into several stages: activation and proliferation of satellite cells, which are the muscle stem cells, differentiation or fusion of the muscle stem cells, maturation of the newly formed muscle fibers and the remodeling of muscle fibers. This process requires high levels of protein synthesis, proper mRNA translation, and energy consumption. Different signaling pathways underlie these processes, such as AMP-activated protein kinase (AMPK) signaling, which is regulated by CaMKII and liver kinase B1 (LKB1) [117,118]. In C3KO mice, during muscle regeneration after CTX injection, muscle fiber growth is arrested due to increased AMPK phosphorylation, inhibition of mTORC1, and energy shortage [71]. Regeneration is one of the most energy-consuming cellular processes, and therefore, C3KO mice fibers are not able to activate genes necessary to adapt to a new situation [119,120]. Moreover, abnormal sarcomere organization in C3KO mice may also contribute to impaired muscle regeneration [69].

Interestingly, in regenerating C3KO muscles [71], as well as in LGMDR1 biopsies [121], a similar miRNA pattern has been reported to the one described in muscles with impaired myofiber repair/regeneration and subsequent fibrosis, showing increased Pax7 expression and downregulation of the muscle specific miRNAs miR-1, miR-133a, and miR-206. These miRNAs, also known as dystromirs, are involved in myogenesis by promoting muscle proliferation (miR-133a) and differentiation (miR-1 and miR-206) [122]. Downregulation or inhibition of miR-1 and miR-206 is associated to increase in the proliferation of satellite cells and Pax7 expression in vivo [123,124]. On the other hand, miR-133a is involved in the maintenance of adult skeletal muscle structure, function, bioenergetics and myofiber identity [125]. Remarkably, mir-1, miR-206, and miR-133a have been proposed as disease biomarkers for Duchenne muscular dystrophy [122].

Finally, MyoD modulation by CAPN3 has been demonstrated in murine cell cultures, which signals CAPN3 as a potential player during muscle regeneration [126]. Moreover, a defective fusion of C3KO myoblast has been described in vitro, due to an accumulation of the β-catenin-M-cadherin complex at the membrane [127]. CAPN3 regulates the localization of β-catenin [127]. At the same time, FRZB, through the inhibition of the Wnt pathway, may prevent the translocation of β-catenin to the nucleus [104]. Interestingly, upregulated FRZB expression has been found in LGMDR1 muscle samples [104]. Therefore, CAPN3 seems to be involved in the fusion and maturation processes of myogenic cells.

### 4.6. Myoapoptosis

Several studies have reported apoptotic myonuclei in muscle samples from LGMDR1 patients [91] and Capn3^−/−^ CAPN3 knockout mice [101]. Conversely, similar studies performed in the C3KO mice fail to detect apoptotic nuclei in muscle fibers [69]. Differences in Ca^2+^ buffer capacity or protective response against oxidative stress may account for the lack of apoptotic nuclei in this mouse model of LGMDR1.

In LGMDR1, apoptosis may be triggered through several pathways (see Figure 5). First, CAPN3 activates NFκB in a Ca^2+^ dependent manner, through degradation of the NF-κB inhibitor IκBα [101]. Activation of NF-κB in the skeletal muscle may lead to protein degradation, inflammation, and fibrosis [128], but it also may promote muscle cell survival under certain conditions [129,130]. This seems to be the case in LGMDR1 since previous works have shown that CAPN3 regulates the expression of NF-κB-dependent survival genes to prevent apoptosis in skeletal muscle, such as c-FLIP [129]. In particular, the expression of c-FLIP, a master anti-apoptotic regulator downstream NF-κB signaling pathway, is downregulated in human myotubes and mouse muscle deficient for CAPN3 [68]. Deregulations in the NF-κB pathway could be part of the mechanism responsible for the muscle wasting resulting from CAPN3 deficiency.

On the other hand, Cn also plays a significant role in apoptosis through the dephosphorylation of proteins involved in the apoptotic pathway, such as caspase 9. Phosphorylation of caspase 9 by Akt pathway results in caspase 9 inhibition, while Cn triggers caspase activity via dephosphorylation [131]. Third, Cyt-c leakage from mitochondria promotes apoptosome generation in the cytosol, which activates caspase 9 and the downstream caspase cascade, and may result in myoapoptosis [113]. Interestingly, a cytosolic Cyt-c localization has been reported in LGMD2A muscle biopsies [91].

Lastly, sustained SR stress, elevated intracellular calcium, and oxidative stress, all of which underlie LGMDR1 pathology, may also lead to apoptosis through caspase activation. Therefore, further studies are needed to elucidate the molecular mechanisms that trigger the apoptotic pathway and muscle waste in LGMDR1. These findings may be highly valuable for identification of novel therapeutic candidates.

## 5. Therapeutic Approaches for LGMDR1

Similar to other hereditary disorders, there are currently no effective therapies to treat LGMD. Moreover, treatments for these diseases have mostly been symptomatic so far. However, new treatments, both non-disease- and disease-specific, are emerging for LGMD [132]. Among non-disease-specific treatments, both strength and aerobic exercise training are beneficial for LGMD. In particular, strength training improves strength in LGMDR1, although improvement generally seems smaller than with endurance training [133,134]. One proposed pharmacological approach is to increase muscle mass by enhancing positive regulators of muscle growth, or by inhibiting negative regulators, such as myostatin. In this regard, in the Capn3^−/−^ mouse model of LGMDR1, treatment with AAV-carrying a mutant myostatin propeptide with an inactive C-terminal domain has shown increased muscle mass and improved force generation [135]. On the other hand, a Phase I/II trial with MYO-029, a recombinant human antibody that inhibits myostatin activity has shown good tolerability but minimal improvement in the muscle strength and the pathology of patients with LGMD [136].

Although a meaningful effort has been made in all these areas to address the challenge of developing therapies for LGMDR1, to date, most groups are focused on correcting the primary genetic defect through the use of adeno-associated virus (AAV) gene therapy, transcriptional modification approaches (exon skipping and induction of stop codon read-through) or cell therapy (stem cells and induced pluripotent stem cells) [141]. In the particular case of LGMDR1, there are currently few ongoing therapeutic approaches, and so far, they are only showing a moderate efficacy. Therefore, there is a need for developing new therapeutic strategies directed toward alternative targets of the disease, such as the ones summarized in Figure 2. To date, in the LGMDR1 mouse model, transfer of the AAV-mediated CAPN3 gene has shown safety and efficacy, resulting in significant myopathology amelioration [137]. Moreover, gene correction in LGMDR1 patient-specific iPS cells has been successfully achieved. Those genetically corrected iPS cells might be evaluated in vitro and in vivo once differentiated into skeletal muscle progenitors, in order to address the efficient restoration of full-length CAPN3 [140]. Finally, Sarepta, in association with Nationwide Children’s Hospital, has recently announced a gene therapy program using the AAVrh74 vector, designed to replace CAPN3 in the skeletal muscle via systemic administration. AAVrh74 has a robust affinity for muscle cells, with a relatively low level of pre-existing immunity [142]. This vector has been previously used in other gene therapy programs targeting Duchenne Muscular Dystrophy and five other LGMDs [143,144]. All the therapeutic approaches for LGMDR1 described above are summarized in Table 1.

## 6. Future Directions and Conclusions

The role of Ca^2+^ dysregulation as a pathological mechanism underlying LGMDR1 is increasingly becoming accepted. Here, we have reviewed different participants disrupted in LGMDR1 that regulate Ca^2+^ homeostasis. Several of these Ca^2+^-handling proteins may be interesting candidates for therapeutic approaches for calpainopathies. In this line, treatment with RyR stabilizers has shown an improvement of muscle function in mouse models of Duchenne and LGMDR4 muscular dystrophies [116,145]. Thus, it would be interesting to test these compounds as well as other therapeutic approaches targeting Ca^2+^-handling proteins compromised in LGMDR1 such as CaMKII and SERCAs. SERCA overexpression could be achieved through AAV-mediated gene therapy [146,147], but several drugs may also prove useful in increasing SERCA expression or function, including adrenoreceptor blockers, adrenergic agonists, hormones, glucocorticoids, natural antioxidants, and small molecule SERCA activators such as CDN1163 [58,148,149]. Alternatively, reduction or down-regulation of SERCA inhibitors SLN [150] and PLN [151] could be considered. Finally, inhibition of SERCA degradation through the ubiquitin-proteasome system has been shown to rescue SERCA1 function in a cellular model of Brody disease [152], and therefore, it might also have therapeutic potential for LGMDR1.

Several therapeutic approaches targeting mitochondrial dysfunction and oxidative stress have been shown to improve muscle function in different dystrophic mouse models and could therefore also be effective in ameliorating LGMDR1 phenotype. These therapies include cyclosporine A and cyclophilin D inhibitors (Debio 025) targeting mitochondrial PTP opening, pargyline for the excessive accumulation of reactive oxygen species and overexpression of PGC1α for mitochondrial dysfunction in post-necrotic dystrophic muscles [153].

While genetic correction of CAPN3 is so far one of the most promising therapeutic approaches aiming to cure LGMDR1, there is still much work to be done before gene therapy is available for these patients. Indeed, these therapies need to overcome several hindrances in LGMDR1 patients, including potential immune response resulting from AAV or CAPN3 introduction, as well as efficient delivery of viral vectors to affected muscles without off-target effects. Moreover, it should be taken into account that not every LGMDR1 patient may qualify for this kind of therapy. In this context, therapeutic approaches targeting key Ca^2+^-handling proteins in LGMDR1 may help ameliorate dystrophic progression in LGMDR1, since, as discussed in this review, Ca^2+^ dysregulation seems to play a central pathogenic role in this disease. In any case, further studies of the different pathogenic mechanisms underlying LGMDR1 are essential for helping us understand the many elusive functions of CAPN3 in the skeletal muscle.

## Figures and Tables

**Table 1:** Therapeutic strategies for limb-girdle muscular dystrophy recessive 1 (LGMDR1).

Therapy	Clinical-Pharmacological Use	State	Comments	Ref.
**Pharmacological Therapy**				
MYO-029	Myostatin human recombinant neutralizing antibody	Competed I/II trial	Minimal improvement in muscle strength	[136]
**Gene therapy**				
AAV-delivered mutant myostatin propeptide	Prevention of the cleavage of myostatin propeptide	Preclinical	Increased muscle mass and force generation in mice	[135]
AAV-mediated transfer of calpain 3	Increase of calpain 3 expression and function	Preclinical	Rescue of the contractile force deficits in mice	[137]
Plasmid DNA	Increase of calpain 3 expression and function	Active project		[138]
AAVrh74 vector	Increase of calpain 3 expression and function	Active project	Systemic delivery to muscle	[139]
**Cell therapy**				
iPSC	Increase of calpain 3 expression and function	Active project		[140]

## Abbreviations

AAV	Adeno-associated virus
Akt	Protein kinase B
AldoA	Aldolase isoform A
AMP	Adenosine monophosphate
AMPK	AMP-activated protein kinase
ATP	Adenosine triphosphate
ATP5D	ATP synthase subunit delta
C3KO	Calpain 3 knockout mouse
CaM	Calmodulin
CAMK	Ca^2+^/calmodulin-dependent protein kinase family
CAMKII	Ca^2+^/calmodulin-dependent protein kinase type II
CAPN1	Calpain 1
CAPN2	Calpain 2
*CAPN3*	Human calpain 3 gene
CAPN3	Human calpain 3 protein
Capn3^−/−^	Calpain 3 knockout mouse
CBSW	Calpain-type beta-sandwich
c-FLIP	Cellular FLICE inhibitory protein
CK	Creatine Kinase
Cn	Calcineurin
CSQ	Calsequestrin
CTX	Cardiotoxin
Cyt-c	Cytochrome C
DHPR	Dihydropyridine receptor
ECC	Excitation-contraction coupling
FRZB	Frizzled Related Protein
HDAC	Class II histone deacetylases
IκBα	Nuclear factor of kappa light polypeptide gene enhancer in B-cells inhibitor, isoform alpha
iMOC	Intermolecular complementation
iPSC	Induced pluripotent stem cell
IS1	Insertion sequence 1
IS2	Insertion sequence 2
LGMD	Limb girdle muscular dystrophy
LGMD2A	Limb girdle muscular dystrophy type 2A, renamed LGMDR1
LGMD2B	Limb girdle muscular dystrophy type 2B, renamed LGMDR2
LGMDD4	Limb girdle muscular dystrophy dominant 4
LGMDR1	Limb girdle muscular dystrophy recessive 1, caused by mutations in CAPN3
LGMDR2	Limb girdle muscular dystrophy recessive 2, caused by mutations in dysferlin
LGMDR4	Limb girdle muscular dystrophy recessive 4, caused by mutations in β-sarcoglycan
LKB1	Liver kinase B1
MAM	Mitochondria-associated sarcoplasmic reticulum membrane
MARP-2	Muscle Ankyrin Repeat Protein-2
MEF2	Myocyte enhancer factor-2
miRNA	Micro ribonucleic acid
mRNA	Messenger ribonucleic acid
mTOR	Mammalian target of rapamycin
mTORC1	Mammalian target of rapamycin complex 1
MyoD	Myoblast determination protein 1
NCX	Na^+^/Ca^2+^ exchanger
NCX3	Na^+^/Ca^2+^ exchanger isoform 3
NFAT	Nuclear factor of activated T-cells
NF-κB	Nuclear factor kappa-ligjht-chain-enhancer of activated B cells
NS	N-terminal addition sequence
Pax7	Paired box protein 7
PC1	Protease core subdomain 1
PC2	Protease core subdomain 2
PEF	Penta E-F hand
PGC1α	Peroxisome proliferator activated receptor gamma coactivator 1 alpha
PKA	Protein kinase A
PLN	Phospholamban
PMCA	Plasma membrane calcium ATPase
PTP	Permeability Transient Pore
ROS	Reactive oxygen species
RyR	Ryanodine receptor
RyR1	Ryanodine receptor isoform 1
SERCA	Sarco/endoplasmic reticulum Ca^2+^-ATPase
SERCA1	Sarco/endoplasmic reticulum Ca^2+^-ATPase isoform 1
SERCA2a	Sarco/endoplasmic reticulum Ca^2+^-ATPase isoform 2a
SLN	Sarcolipin
SOD	Superoxide dismutase
SR	Sarcoplasmic reticulum
T-tubule	Transverse tubule
UPR	Unfolded protein rsponse
Wnt	Wingless-related integration site
